# Enhanced Adhesion of Synthetic Discs with Micro-Patterned Margins

**DOI:** 10.3390/biomimetics7040202

**Published:** 2022-11-18

**Authors:** Weimian Zhou, Xuan Wu

**Affiliations:** 1 Institute of Intelligent Machines, Hefei Institutes of Physical Science, Chinese Academy of Sciences, Hefei 230031, China; 2 Science Island Branch, Graduate School of USTC, Hefei 230026, China; 3 Robotics Laboratory, China Nanhu Academy of Electronics and Information Technology, Jiaxing 314002, China

**Keywords:** hillstream loach, bioinspired disc, adhesion enhancement, hierarchical structures

## Abstract

Many aquatic creatures in nature have non-cooperative surface scaling abilities using suction organs; micro-/nano-scale structures found in different parts of the organs play an important role in this mechanism. Synthetic bioinspired suction devices have been developed, but the mechanisms of bioinspired suction system need further investigation. This paper presents the development of a synthetic adhesive disc inspired by the hillstream loach. The microscopic structures involved in adhesion of the hillstream loach were investigated. Bioinspired suction discs were designed with single-level or hierarchical micropatterned margins. Micro three-dimensional (3D) printing and micro electromechanical system (MEMs) technology were utilized in the fabrication of the discs, and the adhesion performance was tested on substrates with different roughness values. The engaging and disengaging processes of the margin were simulated by carrying out a peeling test on a submerged substrate. The interactions between the liquid film and the microstructures were observed using fluorescence microscopy. The enhanced adhesion forces due to the synergy of the hierarchically micro-patterned margin and the disc cavity were duplicated in the synthetic adhesion system.

## 1. Introduction

There are many aquatic creatures in nature that have non-cooperative surface attachment and scaling abilities. For example, octopuses can use suction cups on their arms to crawl through the sea and catch their prey [1]. Remora can ‘hitchhike’ in the sea, using adhesive discs on their backs to attach to sharks [2]. Clingfish can use discs on their papillae for attachment in intertidal areas [3]. Hillstream loaches use their entire bodies as a suction system [4] and rely on this system to attach to submerged surfaces, resisting the pull of torrents. Generally, surfaces in aquatic environments, whether totally submerged or not, are prone to be slippery and uneven. This is due to many factors including the water film and surface fouling caused by bacteria, algae and invertebrates growing on them. Although most aquatic creatures cannot adhere to such substrates with sufficient shear flow resistance, those mentioned above are able to grasp food and stay on rough surfaces through their unique adhesion mechanisms: sucker-like adhesive generating system. The functions and structures of their adhesion organs have attracted researchers looking to develop synthetic aquatic adhesion systems.

Conventional suckers function using the pressure differential between the inside and outside of the cavity, both in atmospheric and aquatic environments. The main source of adhesion of natural sucker-like adhesion organs is similar to that of conventional suckers [1,5,6]; however, micro-/nano-scale structures found in different parts of the organs also play an important role in the system, despite the different types and forms of natural discs [7,8,9,10,11,12]. The suction cups covering octopus tentacles have microscale protuberances in the cavity. These protuberances cause liquid to flow into the cavity, which creates additional capillary forces to supplement the adhesion force, increasing the adhesion strength of the suction cups [11]. The adhesive discs on the back of remora consist of several rows of macroscale parallel lamellae, which increase the remora’s resistance to slippage and enhances friction to maintain attachment to a moving host [10,12]. In addition, a typical multiscale combination is the ’fin-ray-papillae’ system, which is found in the clingfish and hillstream loach. For clingfish, the large number of papillae on the disc margin can enable attachment to rough surfaces and increase shear strength, which causes a delay in the failure of adhesion and increases the attachment force [13]. Needle-like unculi on the fins of the hillstream loach have a multi-layered structure, with a first layer of hexagonal epithelial cells and a second layer of elongated cone-shaped setae. The disc margin is used to fix and seal its perimeter and contributes less to the adhesion force; it works mainly by improving surface adaptation and increasing wet friction [3,7]. This fin–ray–setae system is thought to functionally adapt to surfaces with varying roughness and enhance reversible adhesion [7,14,15].

Inspired by these organisms, researchers have also designed different types of bioinspired devices and tested their performance. Wang et al. [6] introduced a remora-inspired disc prototype equipped with carbon fiber spinules, which can attach to different surfaces and generate a pull-off force 340 times greater than the weight of the prototype. Sandoval et al. [16] developed an artificial suction disc inspired by clingfish, and correlated the effect of bioinspired features to adhesion performance. Tramacere et al. [17] developed the first passive prototypes of artificial suction cups made of silicone; the prototype was equivalent to octopus suckers in terms of sizes and anatomical proportion. Zhang et al. [18] designed and fabricated an artificial adhesive disc with hexagonal pad-like microstructures; the proposed disc could offer possible solutions for the design of adhesion systems for underwater robots.

Despite the approaches mentioned above, the mechanism of the bioinspired suction system needs further investigation, because the engaging/disengaging procedure of the system involves interactions between the compound suction organ and the non-cooperative surfaces. Tramacere et al. [11,19] proposed that macroscale suckers with hair-structures may exhibit sufficient suction for adhesion on contact substrates. Barnes et al. [20] suggested that the non-Newtonian properties of a thin layer of mucus may play a significant role in adjusting wet adhesion. Persson et al. [21] modeled the attachment and detachment process of the toe pads on contact surfaces based on capillary bridges. Ditsche-Kuru et al. [22] suggested that mayfly larvae can achieve effective underwater adhesion through an interlocking mechanism. However, the role of the multilevel hierarchical structure has not been investigated thoroughly. How the hierarchical micro/nano-structure of the discs’ margin help enhance the adhesion, especially on submerged or moist substrates, remains to be determined. A bioinspired adhesive disc with hierarchical microstructures has not been developed, and the mechanical behavior of proposed discs remains to be explored. In addition, the influence of bioinspired features (i.e., microstructure scale and margin material) on the adhesion performance of discs needs to be further investigated. Furthermore, the hierarchical microstructures and their cooperative actions on interfacial liquid have been ignored; the characterization of liquid flow during disc detachment has never been observed.

In this study, inspired by the ability of the hillstream loach to attach to slippery rocks, we developed a synthetic adhesive disc that functions on rough and wet surfaces. We first investigated the microscopic structures involved in the adhesion mechanism of the hillstream loach using scanning electron microscopy (SEM). Then, we designed a bioinspired suction disc with a hierarchical microstructure in the margin to create a hillstream-loach-inspired lip. We fabricated the bioinspired lip using micro three-dimensional (3D) printing and micro electromechanical system (MEMs) technology, and tested its pull-off force on substrates with different roughnesses. Finally, we simulated the engaging and disengaging process of the lip by carrying out a peeling test on a submerged substrate. The interactions between the liquid film and the microstructures were observed using fluorescence microscopy. By duplicating the biological hierarchical structures, we interpreted the role played by the hierarchically micro-patterned lip to enhance the adhesion forces from the perspective related to capillarity and Stefan adhesion. We experimentally verified the effects of microstructure shape and scale, surface roughness, and margin material on the adhesion force of the bioinspired disc. Finally, a pull-off experiment was performed to characterize the peeling behavior of the bioinspired disc margin, and to illustrate the function of the microstructure in the dynamic peeling of disc’s margin from the substrate.

## 2. Materials and Methods

### 2.1. Bioinspiration and Design

**Figure 1:**
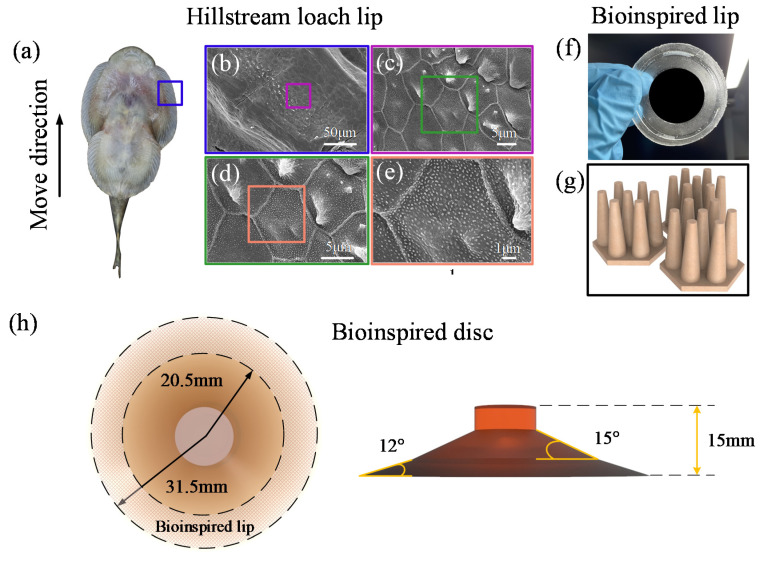
Structure of the hillstream loach biological disc and bioinspired disc: (**a**–**e**) morphology of the structure of the lip of the hillstream loach disc. Blue frame represents the fins of the hillstream loach, purple frame represents the hexagonal epithelial cells, green frame represents a single hexagonal pillar, and orange frame represents the setae; (**f**) synthetic bioinspired lip; (**g**) bioinspired hierarchical microstructure; (**h**) bioinspired disc.

Hillstream loaches (*Beaufortia kweichowensis*) with a total length of about 80 mm were purchased from a commercial supplier (Figure 1a). They were fed in a fish tank that contained rocks and aquatic plants. Fish feed was supplied every day. Fresh lake water was changed each week. Only healthy hillstream loach individuals were used in the experiments.

We observed the morphology of the disc margin of the hillstream loaches (Figure 1b–e) and found hexagonal epithelial cells on the biological disc margin. The length of the hexagon side is about 5 µm. Atop the pillars, there are finer setae with a diameter of about 200 nm. When engaging the rough substrates, hierarchical fibrils on the hillstream loach fins may interlock with surface asperities. This can help seal the margin of the biological disc, as well as enhance tangential friction. Therefore, the superior adhesion capability of the hillstream loach on rough surfaces can also be attributed to the conformance of the margin to the rough surface by hierarchical microstructures. To verify the effect of the hierarchical microstructure on biological discs, we designed a bioinspired lip ring (Figure 1f) which had hierarchical structures (Figure 1g). The primary structure is a hexagonal pillar array; the secondary structure is a cone protrusion array. A disc equipped with the bioinspired lip, as shown in Figure 1h, was prepared for performance testing. The specimen has an inner diameter of 20.5 mm, an outer diameter of 31.5 mm, and a height of 15 mm.

**Figure 2:**
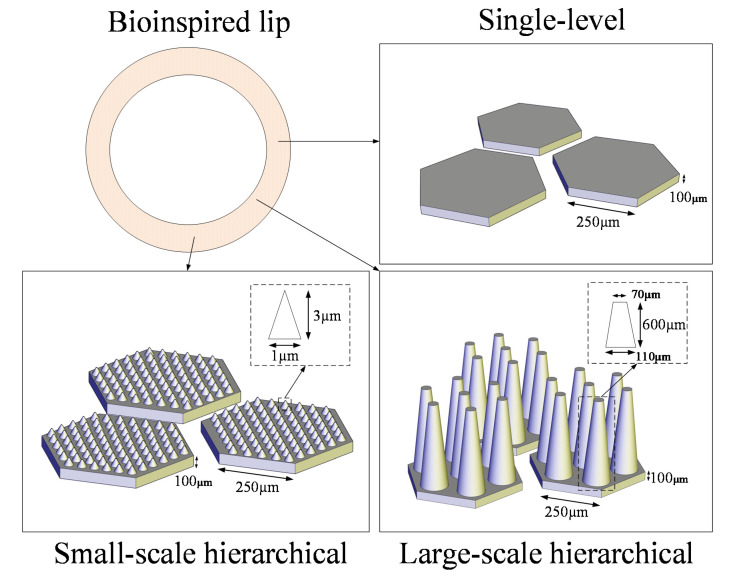
Schematic of the bioinspired microstructure.

We designed three different types of bioinspired microstructures on the lip (Figure 2). The single-level structure includes hexagonal pillar arrays with side lengths of 250 µm and heights of 100 µm. The hierarchical structure utilizes the same hexagonal pillar array as the primary structure, atop which micro-fibers with variable dimensions are distributed. The small-scale secondary structure utilizes a cone-shaped pillar array, whose radius is 1µm and height is 3 µm. The large-scale secondary structure utilizes truncated cone-shaped pillars. The top and bottom diameters of the micro-pillar are 70 µm and 110 µm, respectively, and the height is 600 µm.

**Figure 3:**
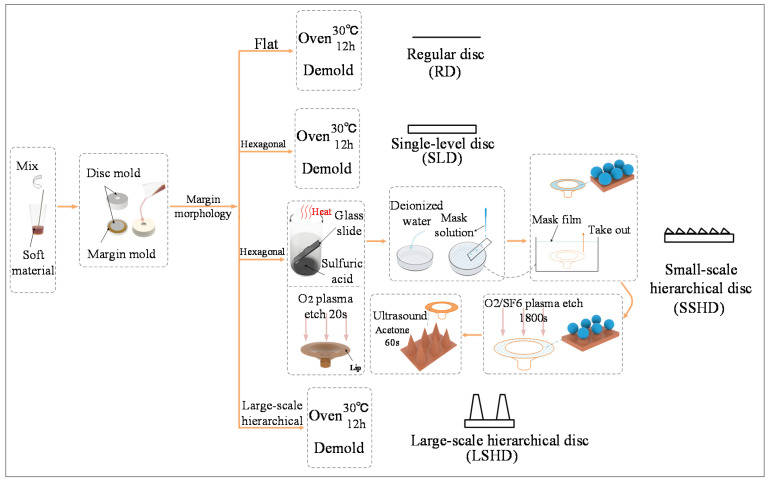
Manufacturing process of the microstructure.

Four types of discs were fabricated, including the single-level disc (SLD), the small-scale hierarchical disc (SSHD), the large-scale hierarchical disc (LSHD), and the regular disc (RD), which was for comparison. The manufacturing process of the discs is shown in Figure 3. Generally, it involved the fabrication of the cavity and the lip’s microstructure; different methods were used for different scales. In the fabrication, the first step is to evenly mix and stir the soft liquid materials (VytaFlex30, Smooth-On, Macungie, PA, USA). The RD, SLD, and LSHD were fabricated using a set of molds, consisting of a cavity mold and a margin mold. The cavity molds of the RD, SLD, and LSHD, and the margin mold of the RD, were fabricated using conventional 3D printing, while the margin molds of the SLD and LSHD were fabricated using microscale additive manufacturing (nanoArch^®^ S140, BMF, https://www.chem-on.com.sg/process-and-manufacturing/bmf-microarch-s140-micro-3d-printer, accessed on 1 November 2022). By pouring the liquid mixture into the assembled molds, the three discs’ prototypes were obtained after curing in the oven at 30 °C for 12 h and MEMs technology (Figure 3). 

To fabricate the SSHD, an extra step using MEMs technology was added, based on the methods for RD. By molding and casting polydimethylsiloxane (PDMS) (Sylgard 184, Dow Corning, Midland, MI, USA), we first fabricated an independent disc margin with a hexagonal morphology the same as the SLD. Then, the margin was placed into a plasma cleaning machine (PDC-32G, Harrick, Ithaca, NY, USA) to etch for 20 s, followed by being submerged into a Petri dish filled with deionized water. The purpose of this step is to increase the hydrophilicity of the disc margin without destroying the surface hexagonal microstructure [23,24,25]. A glass slide was put into the beaker filled with boiled concentrated sulfuric acid to perform temporary hydrophilic modification. Chosen as the etching mask, polymethyl methacrylate (PMMA) spheres (diameter: 1 µm) solution (0.1 g/mL) were continuously dripped onto the glass slide leaning against the edge of the Petri dish with deionized water. The PMMA spheres spread evenly on the water surface when they contact with deionized water. After the dripping, surfactant was dropped on the edge to aggregate the mask. We then removed the disc margin from the Petri dish, whose surface was fully covered with the PMMA spheres mask. The dried disc margin with the mask was placed into the reactive ion etching machine (ME-3A, IMECAS, Beijing, China), and the etching gas was selected as $O_{2}$ and ${\mathrm{SF}}_{6}$ (sccm 5:50) with an etching duration of 1800 s. After the etching process, the mask remnant was removed with PDMS tape. Finally, the soft disc margin was bonded with the disc’s cavity by VytaFlex30 adhesive.

### 2.2. Test Platforms

#### 2.2.1. Mechanical Testing Setup

**Figure 4:**
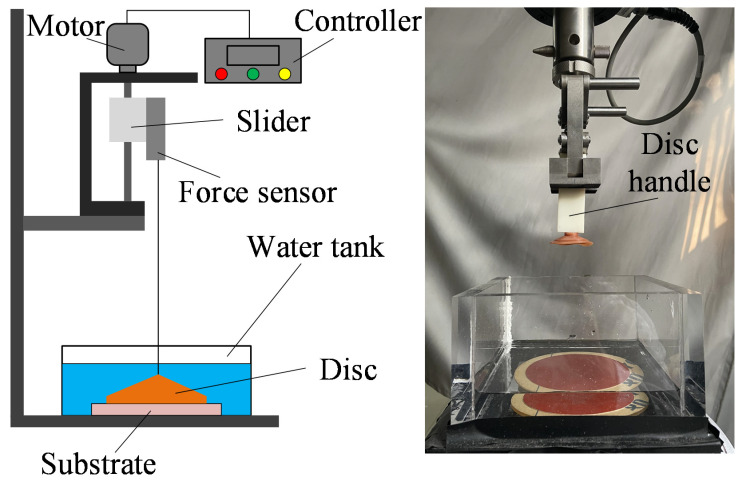
Contact force testing apparatus.

A customized testing apparatus was established for contact force measurement during engagement and disengagement of the disc, which consisted of a universal testing machine (Exceed E44, MTS, Eden Prairie, MN, USA) and an acrylic tank (Figure 4). In the test, specimens were connected to the slider of the universal testing machine via a 3D-printed resin disc handle. The acrylic tank was used to hold water to create an aquatic environment, and testing substrates with different surface roughnesses were fixed on its bottom. The controller regulated the speed of the motor for disc preloading and pull-off. A force sensor was connected to the disc handle for preloading and recording the adhesion force.

#### 2.2.2. Interfacial Observing Setup

**Figure 5:**
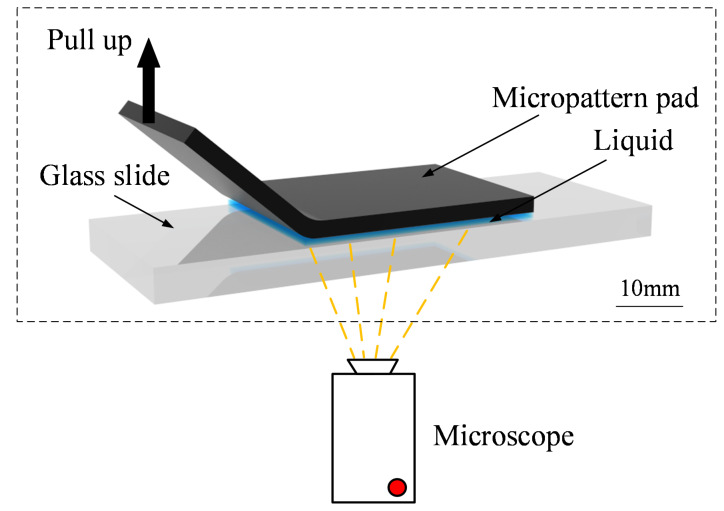
Configuration of the dynamic observation setup.

We used a scanning electron microscope (SEM, EVO18, ZEISS, Oberkochen, Germany) and an optical microscope (DMI3000B, Leica, Wetzlar, Germany) for static surface morphology observed at different scales. For dynamic observation, an experimental setup was established. The setup consisted of a fluorescence microscope (DMI8, Leica) and a glass slide. The fluorescence microscope was invertedly configured (Figure 5), and the glass slide was placed on its stage. The objective lens of the microscope was located underneath the stage. The configuration of the setup was designed to observe the liquid film flow between the micro-patterned pad and the glass slide.

### 2.3. Testing Procedure

#### 2.3.1. Pull-Off Force Testing

We conducted three types of experiments to investigate the performance of the bioinspired adhesive discs during pull-off. In experiment I, the RD, SLD, SSHD, and LSHD were pulled off from a rough substrate (sandpaper with grain size ≈ 10 µm) to investigate the trends in adhesion forces during detachment. In experiment II, the RD, SLD, and LSHD were pulled off from a smooth acrylic surface and different rough substrates (sandpapers with grain size ≈ 5 µm, 7 µm, 10 µm, 18 µm, 22 µm, and 35 µm) to investigate their adaptability to different roughnesses. In experiment III, two specimens of the LSHD with margin materials of PDMS and VytaFlex30 along with the SSHD were pulled off from the rough substrate (sandpaper grain size ≈ 10 µm) to investigate the impact of different surface micro-morphology and textures.

During the tests, slider moved upward at a speed of 500 mm/min. We injected deionized water into the acrylic tank to simulate an immersion circumstance, and the water depth was set as 2 cm. To ensure full adhesion of the disc, 3 N preloading was applied to the disc prototype for 5 s at each engagement.

#### 2.3.2. Interfacial Observation

We carried out interfacial observations of the patterned disc margin in static and dynamic scenarios. In the static observation, optical microscopy was used to observe the morphology of the printed mold first, and we eliminated the mold with structural surface flaws. Micro/nano-scale fibrillary structures were observed using SEM.

In the dynamic observation, we simulated the detachment of the disc margin by peeling it from submerged substrate, which occurred in the desorption of the disc. Before the experiment started, PDMS films with single-level microstructure and hierarchical microstructure on the surface were cut into 2 cm × 1 cm specimens and the PDMS specimens were treated with oxygen plasma for 30s to make the surface hydrophilic. The two surface-treated specimens were lightly placed onto smooth slides, which were then placed on a microscope stage for observation (Figure 5). At the beginning of the experiment, 1 μL of stained deionized water was poured onto one edge of the specimen, ensuring that the contact area between the specimen and the slide was filled with the liquid. The edge of the specimen was then slowly peeled off the substrate with forceps. Due to gravity, the liquid film flowed downwards from the peeled part to the attached part, and a stream was produced. A video of the interaction between the stream and the microscale fibrils in the peel-zone was shot using an inverted fluorescence microscope with an excitation light wavelength of 488 nm.

## 3. Results

### 3.1. Pull-Off Force Results

**Figure 6:**
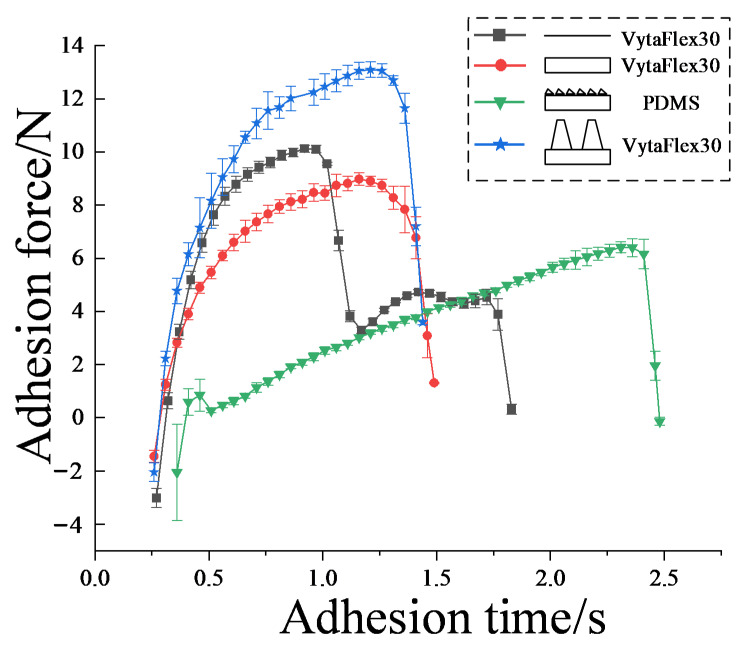
Mechanical testing of discs with different microstructures.

As shown in Figure 6, pull-off force tests were carried out on the SLD, SSHD, LSHD, and RD, and adhesion force date curves with respect to time were recorded during the procedures. The curves for the regular discs show two peaks at 0–1 s and 1.2–1.5 s, and two valleys at 1–1.2 s and 1.5–1.8 s. For discs with microstructures, on the other hand, they all show only one peak and one valley at times after 0.5 s. This indicates that the performance of the regular discs is unstable compared with the bioinspired discs, probably due to the sudden peeling-off of the margin from the substrate. This resulted in a sudden drop in adhesion force, which could also easily lead to complete disc adhesion failure. In contrast, with the gradual increase in pulling force, the peeling of the bioinspired margin was gentle and the adhesion force curves were relatively smooth.

Between 0.5 s and 1.2 s, the adhesion forces of the LSHD, SSHD, and SLD increased by 5 N, 3.7 N, and 2.6 N, respectively. In addition, the maximum adhesion force, or pull-off forces of each type of bioinspired disc, showed a decreasing trend: the pull-off forces for the LSHD, RD, SLD, and SSHD were 13.2 N, 10 N, 9 N, and 6.1 N, respectively. The LSHD and SLD reached their global peaks simultaneously at 1.2 s, while the SSHD reached its global maximum pulling force at 2.3 s; after this time, all values dropped rapidly to 0. The reasons for the different pull-off forces and the three types of discs not detaching at the same time might be due to different microscale morphologies and the different matrices of the margin. Adhesion force is mainly produced by the pressure differential due to cavity deformation; therefore, the margin of the LSHD might be able to resist the highest shear strength to provide highest deformation of the cavity. Simultaneous desorption, on the other hand, is probably due the spontaneous peeling procedure of the margin. The elastic modulus of PDMS (≈5 MPa) is higher than that of VytaFlex30 (≈0.45 MPa); therefore, the deformation of the disc cavity would reach its critical value more slowly, consequently slowing down the speed of the total desorption. 

Despite the differences in the adhesion times of the discs, when each of the discs tested reached their maximum force, they all dropped from their local maxima to 0 within 0.3 s. This indicates that the discs detached within a short period of time when they reached their peak adhesion force. Whether the disc margins were equipped with microstructures or not, they did not extend the maintenance of the maximum adhesion force or prevent the desorption behavior from occurring.

**Figure 7:**
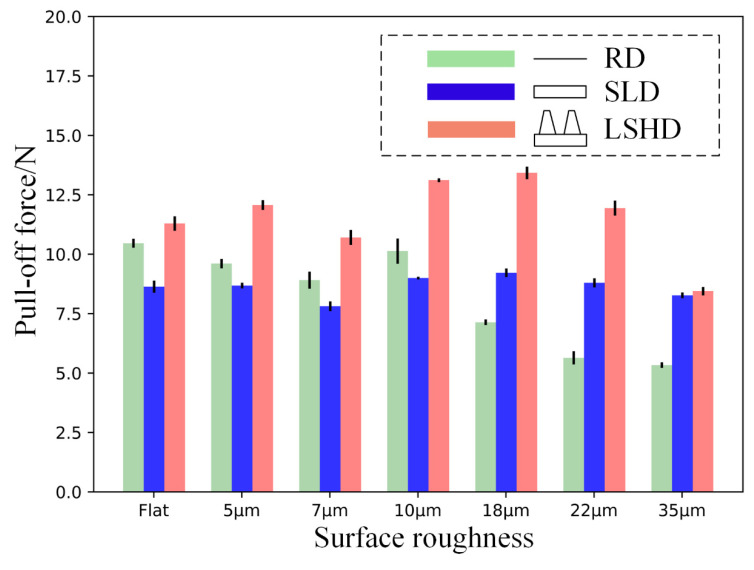
Influence of surface roughness on the pull-off force.

As shown in Figure 7, the pull-off force of the LSHD was consistently higher than that of SLD and RD, regardless of the sandpaper substrate. When the surface roughness increased, the pull-off force of the LSHD tended to increase and then decrease, reaching a maximum value of 13.2 N at a sandpaper grain size of 18 µm. This was due to the hierarchical microstructure which prevented the margin of the disc from peeling and sliding, resulting in large tensile force required to peel the lip away from the substrate. As the surface roughness increased to 35 µm, the maximum pull-off force decreased to a global minimum of 8.6 N. This indicates that the hierarchical microstructure margin still exhibited certain surface adaptability to high surface roughness to ensure the disc seal. However, this seal was weak and required only a small tensile force to peel the disc margin away from the sandpaper. It can be assumed that the contact between the disc and the substrate was not tight at this point and that external liquids and air could easily invade the disc vacuum area, leading to an eventual detachment.

When the contact substrate was relatively smooth (roughness less than or equal to 10 µm), the maximum adhesion force of the SLD was lower than that of the RD, but the difference between them decreased as the roughness increased. This means that as the substrate became rougher, the surface adaptability of the RD became worse and that of the SLD became better. When the disc was applied to a rougher (roughness > 10 µm) substrate, the maximum adhesion force of the SLD exceeded the maximum adhesion force of the RD for the first time, and the differential between the two became higher as the roughness increased. When the surface roughness reached 35 µm, the RD could only resist 5.2 N of pull-off force before detaching from a 35 µm substrate, which suggests that the contact between disc margin and substrate is very vulnerable at this state. In this circumstance, the SLD or LSHD would be a much better choice.

**Figure 8:**
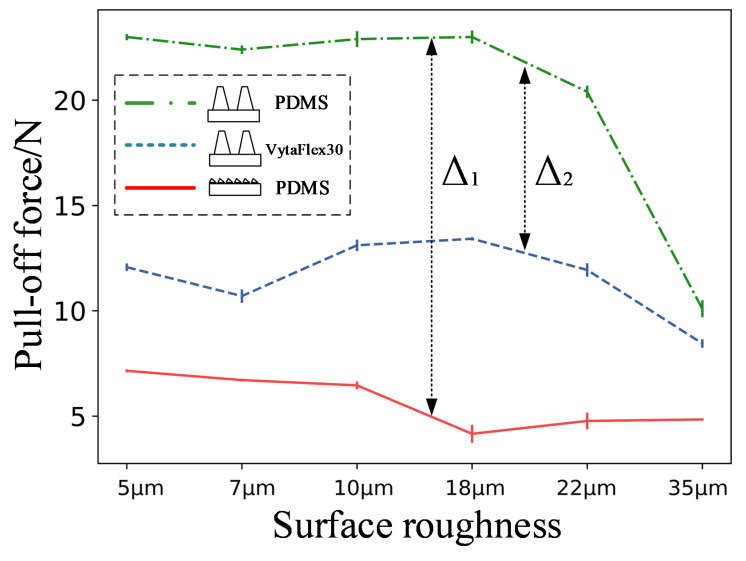
Effect of the material and microstructure shape on the pull-off force.

When the discs were equipped with large-scale hierarchical microstructures made of PDMS, their pull-off force (red dotted line) was consistently higher than those equipped with small-scale hierarchical microstructures made of PDMS (green solid line) and large-scale hierarchical microstructures made of Vytaflex30 (blue dashed line) (Figure 8). This indicates that an optimal combination of surface morphology and margin matrix might enhance the total adhesion performance. Denoting their differentials as $\Delta_{1}$ and $\Delta_{2}$, respectively, it can be seen that $\Delta_{1}$ is consistently higher than $\Delta_{2}$. This indicates that the microstructure morphology had a greater influence on the disc pull-off force than the material properties.

### 3.2. Interfacial Observation Results

#### 3.2.1. Static Observation

**Figure 9:**
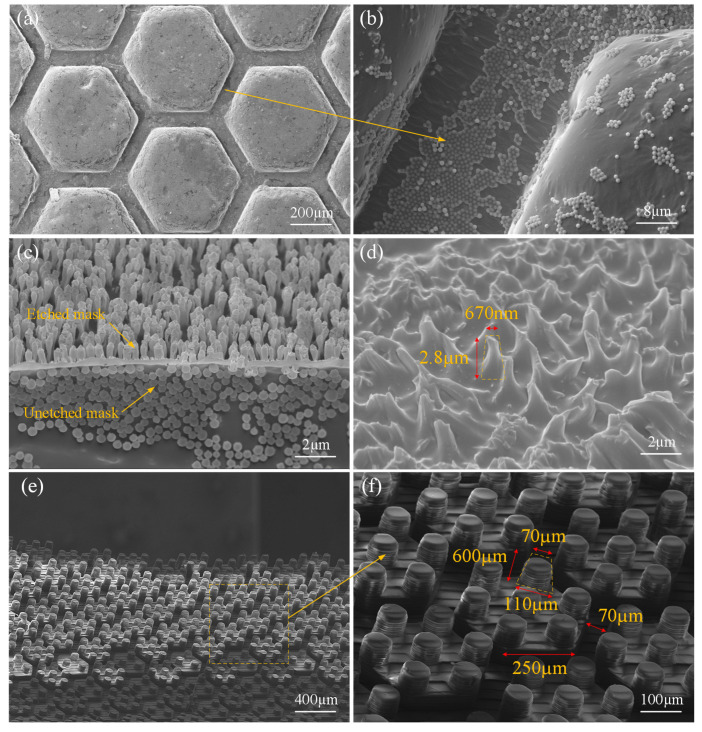
Morphology of the hierarchical structures: (**a**) a hexagonal microstructure substrate covered with a PMMA mask, the yellow arrow represents the boundary of the hexagonal microstructure; (**b**) the distribution of PMMA mask microspheres on the boundary of the hexagonal microstructure and in the groove; (**c**) surface etched by oxygen and sulfur hexafluoride plasma; (**d**) small-scale hierarchical microstructure after removing residual PMMA mask microspheres; (**e**,**f**) large-scale hierarchical microstructure manufactured by 3D printing demolding, the yellow arrow represents the details of the large-scale hierarchical microstructure.

The short-term oxygen plasma etching of a substrate helps a hydrophobic surface to obtain hydrophilicity, without destroying the surface microstructure; thus, the solution can be evenly spread on the substrate without the aggregation of droplets. In addition, the surfactant sodium dodecylbenzene sulfonate (SDBS) can help form a closely packed PMMA sphere monolayer, which plays a critical role in the assembly process. In static observations, it can be seen that masked microspheres formed a lot of molecular self-assembly clusters on the substrate, as shown in Figure 9a. The edges of the hexagonal microstructure were not flat; therefore, part of the masked microspheres slid along the edge into the groove (Figure 9b).

After successful preparation of the closely packed PMMA masks, a monolayer with a gap between the PMMA spheres could be fabricated by ${\mathrm{SF}}_{6}$ plasma reactive ion etching. It can be seen from Figure 9c that inter-sphere distance was produced in etched mask area, leading to an ordered cylinder-like structure. Unetched masks were found on surfaces that were inaccessible to ${\mathrm{SF}}_{6}$ (such as the groove surface), which retained their unetched, spherical structure. After rinsing off the residual PMMA mask on the surface with acetone, a small-scale hierarchical microstructure was obtained (Figure 9d). The small-scale microstructure had a shape similar to a truncated-cone structure, with a height of 2.8 µm and an upper diameter of about 670 nm. Notably, the material of the etched surface was very soft; therefore, the microstructure formed by dry etching is not completely identical with the design.

Our study also observed a large-scale hierarchical microstructure manufactured by 3D printing demolding. The microstructure included a hexagonal base and a circular truncated cone. The upper and lower diameters of the circular cone were 70 µm and 110 µm, respectively, and the height was 600 µm (Figure 9e,f). The aspect ratio and the scale of large-scale hierarchical microstructure were much higher than that of the small-scale hierarchical microstructure.

#### 3.2.2. Dynamic Observation

**Figure 10:**
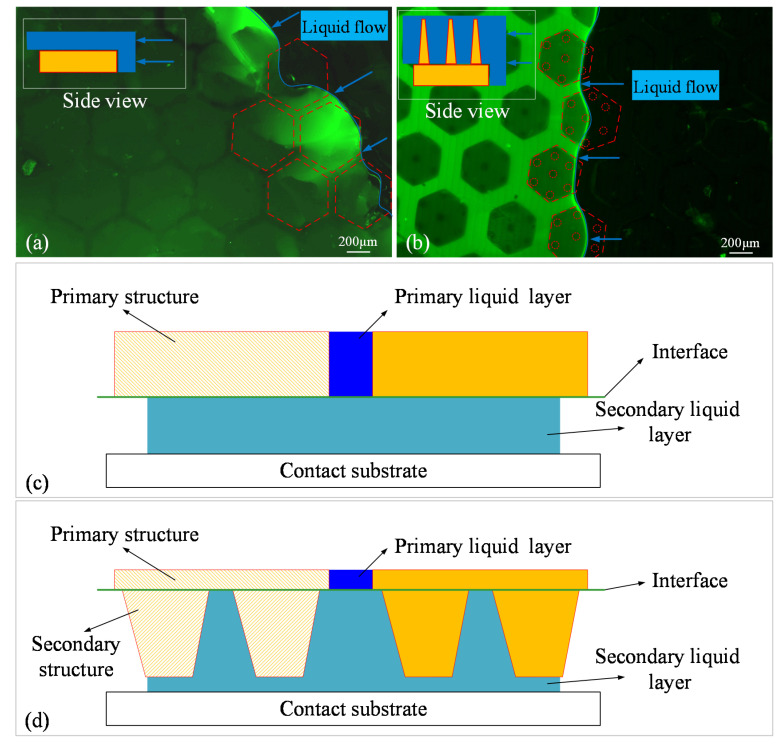
Variation in the contact interface when different lip ring microstructures are peeled from the substrate. A thin liquid film exists between (**a**) the single-level and (**b**) the large-scale hierarchical micropatterned surface and the substrate, respectively; (**c**) Cross-sectional profile of the single-level specimen with different liquid layers; (**d**) Cross-sectional profile of the hierarchical specimen with different liquid layers.

In the dynamic observation, we found that, no matter which specimen was in contact with the acrylic plate, there was always a thin liquid film (green shiny area in Figure 10a,b) between the micropatterned surface and the plate, and neither the primary hexagonal pillar nor the secondary cone-like structure was in direct contact with the substrate. This means that when the disc was being pulled-off, tangential slip mainly occurred in the contact area between the disc margin and the liquid film. In Figure 10c,d, the cross-sectional profile of the two specimens with different liquid layers is marked for clarity. In Figure 10c, the flowing liquid can be regarded as a combination of two layers: the primary layer and the secondary layer. The primary layer is in the distal end from the contact substrate and flows in the grooves between the hexagonal pillars, and the secondary liquid layer is in the proximal end from the contact substrate and flows between the upper surface of the hexagonal pillars and the contact substrate. Liquid layers are similarly defined in Figure 10d, where the secondary liquid layer flows between the surface of the secondary microstructures and the contact substrate. In the fluorescence images, the primary liquid layer is brighter and the secondary liquid layer is darker. As shown in Figure 10a, the fluorescence intensity of the stained water is unevenly distributed in the liquid near the border. This indicates that as the liquid film slides across the upper surface of the hexagonal microstructure, there is local aggregation of the liquid, resulting in an uneven thickness of the liquid film close to the boundary section. However, this liquid aggregation did not occur on the upper surface of the hierarchical microstructure. As shown in Figure 10b, the liquid film also appeared to be bright in some areas and dark in others; however, this difference in fluorescence intensity was not due to liquid film aggregation, because the borders of bright and dark areas were in accordance with the profiles of hierarchical structures in the ventral view. The height of the primary structure in the hierarchical microstructure was 100 µm and the height of the secondary structure was 600 µm, which resulted in the brightness difference.

**Figure 11:**
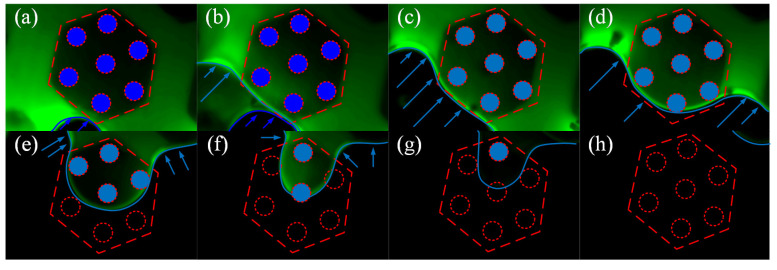
Migration process of liquid film in a single hierarchical microstructure unit: (**a**) The hierarchical fibril was submerged by the liquid film; (**b**) The liquid film began to separate into the primary and secondary layers. The light blue arrow represents the secondary liquid layer boundary, and the dark blue arrow represents the primary liquid layer boundary; (**c**–**g**) Secondary liquid film migrated over a single hierarchical fibril; (**h**) A single hierarchical fibril was totally peeled away from the substrate.

Figure 11 shows the full migration process of the liquid film over a single hierarchical fibril as the specimen is being peeled away from the substrate (Supplementary Materials, Video S1). Originally, the hierarchical fibril was submerged by the liquid and the liquid film had not yet begun to migrate (Figure 11a). Then, the peeling motion led to the spontaneous flow of the liquid due to gravity. As shown in Figure 11b, the liquid film also began to separate into primary and secondary layers, which shared identical interface with the primary and secondary microstructures. The light blue arrow indicates the secondary liquid layer boundary, and the dark blue arrow indicates the primary liquid layer boundary. We then found that the migration of the primary layer continued by flowing through the grooves between the hexagonal pillars, as the boundary advanced (Figure 11c,d). Meanwhile, the secondary layer gradually vanished but remained in the area wrapped by the hexagonal profile (the dash line). Migration of the secondary layer was hindered by the cone-like fibrils, probably due the frictional viscosity between the liquid film and the lateral surface of the fibrils. Figure 11e,f show that the secondary layer lasted until the primary layer had nearly disappeared, but the shrinkage of the secondary layer’s boundary was still hindered by the fibrils. The trend in the shrinkage was finally halted by one single fibril, as shown in Figure 11g, perhaps because the gravity could not provide enough locomotion to overcome the friction with the fibril. The thickness of the liquid film started to decrease; instead, the fluorescence intensity decreased significantly. Figure 11h shows that there was no longer any fluorescence in the screen, indicating that the liquid film had finished migrating over the single hierarchical fibril and the specimen had been peeled away from the substrate.

## 4. Discussion

**Figure 12:**
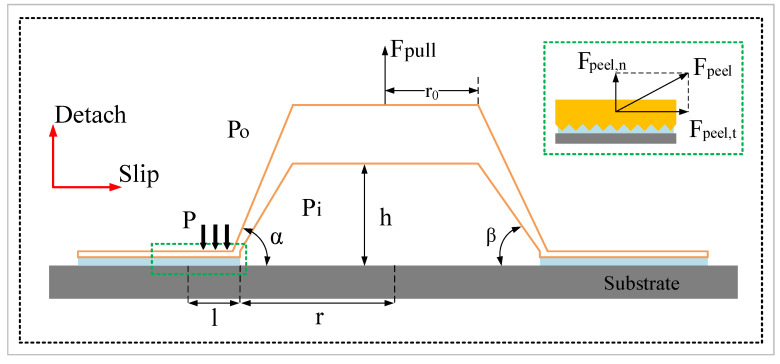
Model of the bioinspired disc.

As has been suggested by Zou et al. [5] and Chuang et al. [7], the adhesive force of the hillstream loach is mainly produced by a combination of the pressure differential and the capillary force; the former is dominant over the latter. However, the adhering process involves more factors acting on each other, and complete adhesion cannot simply be regarded as their summation. A mechanical model of the disc was established, as shown in Figure 12. In the model, the disc was pulled upwards by normal force, $F_{pull}$, and the pressure differential ($P_{o}$−$P_{i}$) between the inside and outside of the disc cavity caused the disc to attach to the substrate. The actual contact area between the disc margin and the substrate was an annular area with a width of l. Persson et al. [26] suggested that the contact pressure between them is constant: p. The margin of the disc was subjected to a tangential peel force, $F_{peel,t}$, and a normal peel force, $F_{peel,n}$, for tangential slip and normal dislodgement, respectively.

### 4.1. Enhanced Adhesion Force Due to Higher Capillary Force

**Figure 13:**
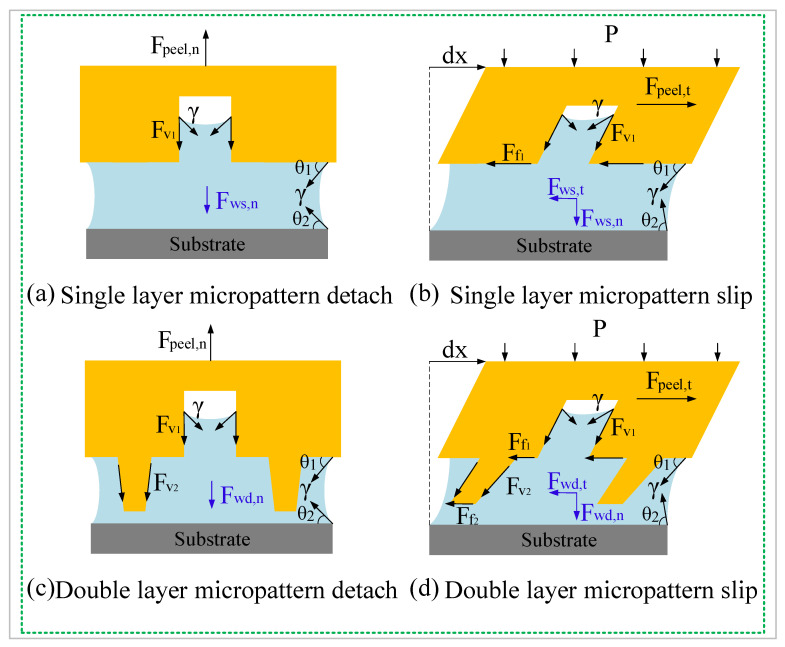
Diagram of the bioinspired margin in contact with the submerged substrate.

In addition to the pressure differential, wet adhesion is a complement of the adhesion force. Figure 13 shows a free body diagram of the bioinspired margin in contact with the submerged substrate. As shown in Figure 13a, when part of the SLD margin is being peeled off from the substrate, the hexagonal pillar is affected by the frictional viscous force, $F_{v1}$, between the liquid and the groove; the surface tension, γ, of the liquid; and the normal component of the wet adhesive force, $F_{ws,n}$, of the liquid. However, when the single-level surface pad slipped laterally, the pad was not only affected by the forces mentioned above, but also by the frictional force, $F_{f1}$, between the liquid and the upper surface of the hexagonal structure and the tangential component of the liquid wet adhesion force, $F_{ws,t}$ (Figure 13b). For the LSHD, when the hierarchical margin was pulled up normally, due to the presence of the secondary microstructures, the margin was subjected to an additional frictional viscous force, $F_{v2}$ (Figure 13c). This extra force was produced by the contact of the liquid and the side wall of the secondary structure, with the direction of the force being downwards along the side wall of the secondary structure. Similarly, the hierarchical fibril was subjected to an additional frictional force, $F_{f2}$, as it slid in the tangential direction, produced by the contact between liquid and the upper surface of the secondary structure, whose direction is opposite to the slip of the margin. Therefore, the secondary structure helped strengthen the wet adhesion effect between the liquid and the disc margin. In addition, after the liquid in the grooves between the primary structures completely drained out, the presence of $F_{v2}$ and $F_{f2}$ enabled the remaining liquid to attach to the array of secondary structures, which also helped to explain the distribution pattern of the liquid film. The sealing effect of the hollow structures could be explained by the increase in fluidic resistance in the grooves between the disc margin and the substrate. The air and liquid in the grooves formed a partial wetting pattern, which helped the disc to maintain a seal, leading to a larger adhesive tenacity in wet conditions. In addition, when the turbulence impacted on the disc, these hollow structures formed many microchannels, which helped to diffuse the impact of the water flow and secured the perimeter of the disc.

### 4.2. Enhanced Adhesion Force Due to the Synergy Effect

**Figure 14:**
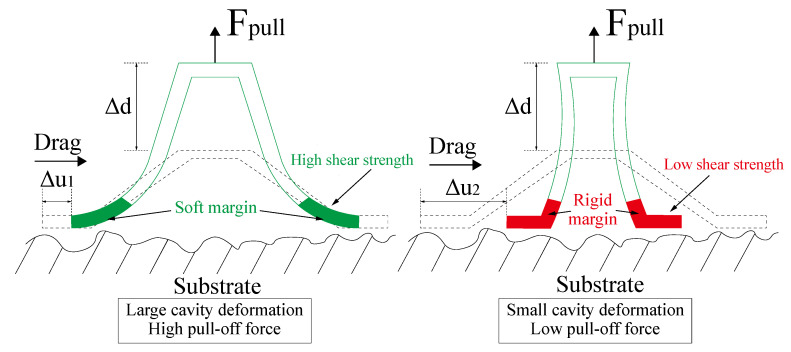
Deformation analysis of different discs.

Schematics of the pull-off procedure of the four disc samples are illustrated in Figure 14. The dashed lines represent the original disc, and the solid lines represent the deformations. In the procedure, the disc were pulled up in the vertical direction for a distance marked as Δd. Spontaneously, the disc margin is dragged inward towards the disc center in the tangential direction, with a distance marked as Δu. Peeling also occurred from the proximal end to the distal of the disc margin with respect to the disc center. The results in Figure 6 indicate that the adhesion force is a function of the pull-off time. The pull-off motion was exerted by the MTS straight upward with prescribed motion function; the four disc specimens exhibited identical displacement in the vertical direction. Ge et al. [27] suggested that elastic deformation of the disc’s cavity will contribute to the pressure differential inside and outside the chamber of the disc. In case of no leakages of air or liquid into the chamber, the shear displacement of the margin edge determined the pull-off force. The total desorption was triggered by the total peeling-off of the margin. Therefore, by studying the curves of the LSHD and SLD, we believe that the margin of the LSHD had higher shear strength than the SLD which resulted in a higher pressure differential for a higher pull-off force. Dynamic observations also verified that the hierarchical fibrils could enhance the shear strength in the submerged circumstance. The peeling, however, occurred at the same time, probably due to the same margin matrices.

The RD and SSHD exhibited two peaks during the procedure. Although their adhesion performances were weakened, the total desorption was delayed. For the RD, we believe that sudden shrinkage of the margin occurred, which lowered the adhesion force but enhanced the adhesion of the margin. For the SSHD, the margin’s shear strength was the lowest, because its shrinking was the earliest. However, the time for total desorption was the longest of the four disc. This phenomenon might be due to the different elastic modulus values of the margin material. The elastic modulus of PDMS (≈5 MPa) is almost tenfold greater than that of VytaFlex30 (≈0.45 MPa); thus, the former is harder to peel from the surface, which is similar to the “peel-zone” phenomenon in the peeling of the adhesive strip tape [28]. Results in Figure 8 also verified this hypothesis.

Overall, the adhesion performance of the bioinspired disc is the synergy of the micro-patterned margin and the disc cavity. The hierarchical morphology enhances the normal and shear adhesion strength of the margin on the liquid layer, which, in turn, increases the cavity deformation, ensuring a considerable pressure differential between the chamber and the atmosphere. A combination of the margin morphology and matrix could be optimized for the best adhesion performance.

## 5. Conclusions

In this study, learning from the hillstream loach, bioinspired discs with different micro-patterned margins were fabricated using 3D printing, etching, and self-assembly methods. The mechanical behaviors of the discs were tested. The influence of the disc margin’s microscale morphology, matrix material, and substrate roughness on the adhesion performance was investigated. Static and dynamic observations were made to investigate the surface morphology and the whole procedure of the liquid film’s migration over the hierarchical fibril during the simulated peeling process. Mechanical models considering the interaction between the disc’s cavity deformation and the margin’s wet adhesion were established to determine the synergy mechanism of the adhesion.

The results show that the proposed bioinspired discs had superior adhesion performance compared with the regular disc, including the increased pull-off force threshold, better adaptability to variable surface roughness, and more stable mechanical behavior in pull-up tests. The secondary cone microscale fibrils acted as barriers to the migration of the fluid film. The hierarchical microstructure prevented the liquid from flowing between the contact interfaces, and the liquid was easily trapped between the secondary cone microstructures, which helped maintain the wet adhesion of the liquid, making the peeling of the margin more difficult. The enhanced wet adhesion due to bioinspired morphologies not only provided wet adhesive forces, but also strengthened the shear resistance of the margin to enhance the total adhesion performance. An optimized combination of factors, including surface morphologies, margin matrix materials, etc., might help to improve the synergy adhesion performance.

Future work will focus on the establishment of a more rigorous model to quantitatively analyze the effects of the factors mentioned above. Synthetic discs for rough and wet surface adhesion will be designed based on the proposed study for possible applications including wall-climbing robotics, gripping devices, etc.

## Data Availability

The data used to support the findings of this study are available from the corresponding author upon request.

## Supplementary Materials

The following supporting information can be downloaded at: https://www.mdpi.com/article/10.3390/biomimetics7040202/s1. Video S1: Migration process of liquid film in a single hierarchical microstructure unit.
